# Pesticide exposure: the hormonal function of the female reproductive system disrupted?

**DOI:** 10.1186/1477-7827-4-30

**Published:** 2006-05-31

**Authors:** Reini W Bretveld, Chris MG Thomas, Paul TJ Scheepers, Gerhard A Zielhuis, Nel Roeleveld

**Affiliations:** 1 Department of Epidemiology and Biostatistics, Radboud University Nijmegen Medical Centre, P.O. Box 9101, 6500 HB, Nijmegen, The Netherlands; 2 Chemical Endocrinology, Radboud University Nijmegen Medical Centre, P.O. Box 9101, 6500 HB, Nijmegen, The Netherlands

## Abstract

Some pesticides may interfere with the female hormonal function, which may lead to negative effects on the reproductive system through disruption of the hormonal balance necessary for proper functioning. Previous studies primarily focused on interference with the estrogen and/or androgen receptor, but the hormonal function may be disrupted in many more ways through pesticide exposure. The aim of this review is to give an overview of the various ways in which pesticides may disrupt the hormonal function of the female reproductive system and in particular the ovarian cycle. Disruption can occur in all stages of hormonal regulation: 1. hormone synthesis; 2. hormone release and storage; 3. hormone transport and clearance; 4. hormone receptor recognition and binding; 5. hormone postreceptor activation; 6. the thyroid function; and 7. the central nervous system. These mechanisms are described for effects of pesticide exposure *in vitro *and on experimental animals *in vivo*. For the latter, potential effects of endocrine disrupting pesticides on the female reproductive system, i.e. modulation of hormone concentrations, ovarian cycle irregularities, and impaired fertility, are also reviewed. In epidemiological studies, exposure to pesticides has been associated with menstrual cycle disturbances, reduced fertility, prolonged time-to-pregnancy, spontaneous abortion, stillbirths, and developmental defects, which may or may not be due to disruption of the female hormonal function. Because pesticides comprise a large number of distinct substances with dissimilar structures and diverse toxicity, it is most likely that several of the above-mentioned mechanisms are involved in the pathophysiological pathways explaining the role of pesticide exposure in ovarian cycle disturbances, ultimately leading to fertility problems and other reproductive effects. In future research, information on the ways in which pesticides may disrupt the hormonal function as described in this review, can be used to generate specific hypotheses for studies on the effects of pesticides on the ovarian cycle, both in toxicological and epidemiological settings.

## Review

Although a substantial amount of research has been conducted to associate occupational exposure to pesticides with fertility problems in men [1-4], studies among women are scarce. One reason may be that exposure to pesticides is higher among men, because men usually apply pesticides whereas women get exposed through re-entry activities only. Another reason may be that fertility in women is more difficult to assess than fertility in men. The ovarian cycle has not been as fully explored as the spermatogenesis in men. Ovarian disorders can be caused by a large variety of factors, such as high levels of physical activity, age, stress, smoking, and caffeine use [5-7]. In addition, exposure to chemicals such as benzene and polychlorinated biphenyls (PCBs) can affect the menstrual cycle [8,9]. There are also indications that exposure to particular pesticides may induce ovarian dysfunction. Recently, Farr *et al. *examined the association between pesticide exposure and menstrual cycle characteristics [10]. They observed that women who worked with pesticides suspected of being hormonally active had a 60–100% increased odds of experiencing long cycles, missed periods, and intermenstrual bleeding compared with women who had never worked with pesticides. In two studies on time-to-pregnancy among female greenhouse workers [11,12], the authors concluded that female workers in flower greenhouses may have reduced fecundability and that exposure to pesticides may be part of the causal chain. In one other study, an increased risk of infertility was observed among women exposed to pesticides or working in industries associated with agriculture [13,14].

Some pesticides may interfere with the female hormonal function and thereby cause negative effects on the reproductive system. Most previous studies focused on interference with the estrogen and/or androgen receptor, but the hormonal function can be disrupted in many more ways through pesticide exposure. The aim of the present review is to give an overview of the various ways in which pesticides may disrupt the hormonal function of the female reproductive system and in particular the ovarian cycle. As adequate exposure assessment studies in humans are scarce for most pesticides, this review will not provide information on dose-response relations nor does it pretend to discuss risk assessment.

### Female fertility

Subfertility is defined as the inability to conceive within 12 months of regular, unprotected sexual intercourse and affects about 10 – 15% of all couples in the Western world [15]. A World Health Organization (WHO) multi-centre study revealed that the problem was predominantly male in 20% of subfertile couples and predominantly female in 38% of the cases, whereas 27% showed abnormalities in both man and woman and no evident cause of subfertility was identified in the remaining 15% [16]. Five types of subfertility disorders are distinguished [17].

#### 1. Male subfertility

Low sperm concentration, reduced motility and/or abnormal morphology of sperm are the dominant causes of subfertility in 20 – 25% of couples [18-20]. Male subfertility is generally expressed as a reduced ability of the female partner to become pregnant.

#### 2. Ovulation disturbances

Problems with ovulation account for subfertility in another 20 – 25% of couples and is thereby a frequent cause of subfertility in women [18]. Ovulation problems present themselves as irregular or absent menstrual periods and can be substantiated through measurement of reproductive hormones.

#### 3. Defects in spermatozoa-cervical mucus interaction

Abnormal cervical mucus may prevent the sperm from reaching the oocyte. Some authors estimate that cervical hostility is a cause of subfertility in 10 – 15% of couples [18,19], whereas others deny its mere existence [20].

#### 4. Tuboperitoneal disorders

Tubal damage and/or obstruction, hydrosalpinx, pelvic adhesions, and endometriosis are the main cause of subfertility in 10 – 30% of couples [18]. In many instances, these problems originate from infections.

#### 5. Unexplained subfertility

Despite advances in the diagnosis of causes of subfertility, inability to conceive remains unexplained in 25 – 30% of fully investigated couples [18-20].

An important factor in female subfertility is age. The risk of subfertility increases from 10% to 30% when women are over 35 years of age. This is of particular importance nowadays, as an increasing number of women delay pregnancy until the age when natural female fertility is in decline, due to a higher number of chromosomal aberrations in the oocytes. Hormonal balance is another important factor in female fertility, in particular regarding the ovarian cycle. Lifestyle factors, including stress, extreme body weight (too low or too high), coffee consumption, diet, and excessive exercise can affect a woman's hormonal balance and subsequent ovulatory pattern. Hormonal imbalance and ovulatory problems are much less often caused by hormonal diseases, such as pituitary gland tumors. But there are indications that endocrine disrupting chemicals, such as PCBs and certain pesticides, can influence the hormonal balance and thus increase the risk of subfertility [21]. In this review, we will elaborate on hormonal function disturbances associated with pesticide exposure.

### Pesticides

**Figure 1:**
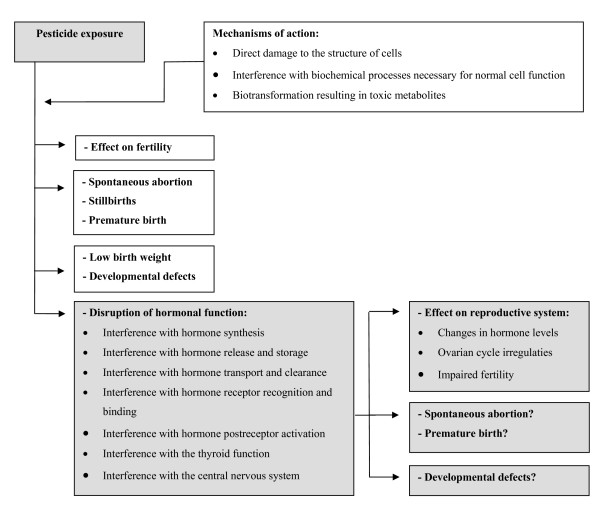
Potential effects of pesticides on female reproduction.

Pesticides are used in agriculture and public health to control insects, weeds, animals, and vectors of disease. The Food and Agriculture Organization of the United Nations (FAO) defined a pesticide as 'any substance or mixture of substances intended for preventing, destroying or controlling any pest, including vectors of human or animal disease, unwanted species of plants or animals causing harm or otherwise interfering with the production, processing, storage, transport, or marketing of food, agricultural commodities, wood, wood products or animal feedstuffs, or which may be administered to animals for the control of insects, mites/spider mites or other pests in or on their bodies' [22]. Next to these intended effects, pesticides may also have adverse health effects for human beings. The main adverse health effects are difficulty in breathing, headaches, neurological or psychological effects, irritation of skin and mucous membranes, skin disorders, effects on the immune system, cancer, and reproductive effects. The manifestation of these effects depends on the type of pesticide and on level and duration of exposure. In this review, we will only focus on potential reproductive effects of pesticide exposure. Pesticides may cause reproductive toxicity through several different mechanisms: direct damage to the structure of cells, interference with biochemical processes necessary for normal cell function, and biotransformation resulting in toxic metabolites (see Figure 1). Reproductive effects that have been associated with pesticide exposure in women are decreased fertility, spontaneous abortions, stillbirth, premature birth, low birth weight, developmental abnormalities, ovarian disorders, and disruption of the hormonal function [23,24]. Pesticides that may disrupt the hormonal function are often called endocrine disrupting chemicals (EDCs), just like other agents with similar mechanisms of action. An ECD may be defined as an exogenous agent that interferes with the synthesis, secretion, transport, binding, action, or elimination of natural hormones in the body that are responsible for the maintenance of homeostasis, reproduction, development and/or behaviour [21,25]. Endocrine disruptors are usually either natural products or synthetic chemicals that mimic, enhance (agonists), or inhibit (antagonists) the action of endogenous hormones [25]. Body burden, dose, timing, and duration of exposure at critical periods in life are important considerations for assessing the risk of an adverse effect of endocrine disruptors. In the next paragraphs, we will review the ways in which pesticides may disrupt the female hormonal function of the reproductive system on the basis of experimental animal studies (*in vivo*) and cell culture studies (*in vitro*), which often provide the first indications of potential reproductive effects (see Table 1). We only describe possible mechanisms of disruption mentioned in the literature to indicate hazards, without judgement about human risks based on dose-response relations.

**Table 1:** Pesticides with known or suspected endocrine disrupting properties, mechanisms and effects on the female reproductive system in experimental animals

**Pesticides**	**Mechanisms**	**Effects in experimental animals**
Alachlor	- binding and activating the estrogen receptor [64] - binding other receptors [81]	- unknown -
Aldrin	- binding and activating the estrogen receptor [55]	- unknown -
Amitraz	- interference with hormone storage and release [37]	- unknown -
Atrazine	- interference with hormone synthesis [29] - binding without activating the estrogen receptor [70,71]	- modulation of hormone concentrations [94,95] - ovarian cycle irregularities [109-112,119]
Diaminochlorotriazine	- binding without activating the estrogen receptor [70,71]	- unknown -
Dicofol	- unknown -	- impaired fertility [127]
Dieldrin	- binding and activating the estrogen receptor [31,59-61] - binding other receptors [31]	- unknown -
Biphenol	- binding other receptors [74,79,80]	- unknown -
Dimethoate	- unknown -	- ovarian cycle irregularities [107,108]
Carbofuran	- unknown -	- ovarian cycle irregularities [113]
Chlordecone	- binding and activating the estrogen receptor [66] - binding other receptors [66]	- ovarian cycle irregularities [103] - impaired fertility [102,104]
Chlordimeform	- interference with hormone storage and release [37]	- impaired fertility [123]
Chlorophenols	- interference with overall metabolic rate [85,86]	- unknown -
Chlorophenoxy acids	- interference with overall metabolic rate [85,86]	- unknown -
DDT analogs	- interference with hormone transport and clearance [47] - binding and activating the estrogen receptor [55-57] - binding other receptors [66,74,76] - interference with the central nervous system [88,89]	- modulation of hormone concentrations [101] - ovarian cycle irregularities [103] - impaired fertility [127]
D-trans allethrin	- binding other receptors [65]	- unknown -
Endosulfan	- interference with hormone storage and release [48] - binding and activating the estrogen receptor [31,58,59] - binding other receptors [81]	- unknown -
Endrin	- binding and activating the estrogen receptor [55]	- unknown -
Fenarimol	- interference with hormone synthesis [26,27] - binding and activating the estrogen receptor [31,62] - binding other receptors [31]	- impaired fertility [27]
Fenitrothion	- binding other receptors [78]	- unknown -
Fenvalerate	- binding and activating the estrogen receptor [65] - binding other receptors [65]	- unknown -
Heptachlor	- interference with hormone synthesis [32]	- modulation of hormone concentrations [92,93] - ovarian cycle irregularities [114] - impaired fertility [93]
Hexachlorobenzene	- unknown -	- modulation of hormone concentrations [96-98] - ovarian cycle irregularities [96-98]
Iprodion	- interference with hormone synthesis [31]	- unknown -
Kepone	- binding and activating the estrogen receptor [54]	- unknown -
Ketaconazole	- interference with hormone synthesis [28,36]	- unknown -
Lindane	- interference with hormone storage and release [49] - binding without activating the estrogen receptor [67-69] - interference with hormone postreceptor activation [82]	- modulation of hormone concentrations [94,95] - ovarian cycle irregularities [68,69] - impaired fertility [125,126]
Linuron	- binding other receptors [74,77]	- unknown -
Malathion	- interference with hormone storage and release [42]	- ovarian cycle irregularities [107,108]
Mancozeb	- unknown -	- ovarian cycle irregularities [116] - impaired fertility [116,122]
Methiocarb	- binding and activating the estrogen receptor [31] - binding other receptors [31]	- unknown -
Methoxychlor	- binding and activating the estrogen receptor [31,50-53] - binding other receptors [74] - interference with the central nervous system [88,89]	- modulation of hormone concentrations [99,100] - impaired fertility [100,102,104,124]
Methomyl	- interference with hormone synthesis [31]	- unknown -
Methyl parathion	- unknown -	- ovarian cycle irregularities [105,106] - impaired fertility [106]
Mirex	- interference with hormone storage and release [48]	- unknown -
Nonylphenol	- binding and activating the estrogen receptor [63]	- unknown -
Organochlorine compounds	- interference with overal metabolic rate [85,86]	- ovarian cycle irregularities [102-104]
Pentachrophenol	- binding and activating the estrogen receptor [63]	- unknown -
Pirimicarb	- interference with hormone synthesis [31]	- unknown -
P,P-DDE	- interference with hormone synthesis [30]	- unknown -
Prochloraz	- interference with hormone synthesis [28] - binding without activiating the estrogen receptor [31] - binding other receptors [31]	- unknown -
Procymidone	- binding other receptors [74,76]	- unknown -
Propamocarb	- interference with hormone synthesis [31]	- unknown -
Propazine	- interference with hormone synthesis [29]	- unknown -
Quinones	- interference with overall metabolic rate [85,86]	- unknown -
Simazine	- interference with hormone synthesis [29] - binding without activating the estrogen receptor [70,71]	- modulation of hormone concentrations [94,95]
Sodium-N-methyl- dithiocarbamate	- interference with hormone synthesis [33]	- ovarian cycle irregularities [33]
Sumithion	- unknown -	- ovarian cycle irregularities [108]
Sumithrin	- binding and activating the estrogen receptor [65,66]	- unknown -
TCDD	- hormone receptor activation [83,84]	- unknown -
3,3',4,4'-tetra chloroazoxybenzene	- unknown -	- ovarian cycle irregularities [117]
Thiram	- interference with hormone synthesis [34] - interference with hormone storage and release [39]	- ovarian cycle irregularities [120]
Toxaphene	- binding and activating the estrogen receptor [58-60]	- unknown -
Triadimefon	- binding and activating the estrogen receptor [62]	- unknown -
Triadimenol	- binding and activating the estrogen receptor [62]	
Vinclozolin	- binding other receptors [72-75]	- unknown -

### Disruption of the female hormonal function

#### 1. Interference with hormone synthesis

All hormones differ in their chemical structure and have a different route of synthesis with innumerable different steps. If one substance or link is disturbed in the chain of hormone synthesis, the hormone may not be produced or may get different properties. Some pesticides, such as fenarimol, prochloraz, and other imidazole fungicides possess the ability to inhibit estrogen biosynthesis through CYP19 aromatase inhibition *in vitro *[26-28], preventing the conversion of androgens to estrogens. Vinggaard *et al. *hypothesized that compounds which can inhibit aromatase acitivity *in vitro *may be able to cause local changes in estrogen and androgen concentrations *in vivo *[26]. Aromatase induction is a physiological mechanism to deactivate xenobiotics, which does not inevitably cause a toxic effect. The pesticides atrazine, simazine, and propazine (2-chloro-triazine herbicides) induce aromatase activity *in vitro *[29]. For p,p-DDE, the induction of aromatase has been demonstrated *in vitro *and *in vivo *[30]. In addition, the pesticides methomyl, pirimicarb, propamocarb, and iprodion can weakly stimulate aromatase activity [31], whereas heptachlor may act as an inducer of testosterone 16-alpha and 16-beta hydroxylases [32].

Thiram, Sodium N-methyldithiocarbamate (SMD), and other dithiocarbamates are known to suppress the dopamine-beta-hydroxylase activity leading to reduced conversion of dopamine to norepinephrine [33-35]. This may lead to changes in hypothalamic catecholamine activity involved in generating the proestrus surge in LH, which stimulates the final stages of ovulation. Goldman *et al. *concluded that SMD is able to block the LH surge and ovulation in rats [33]. Ketaconazole inhibits various enzymes which belong to the CYP450-dependent monooxygenases and also inhibits progesterone synthesis [28,36].

#### 2. Interference with hormone storage and release

Interference with hormone storage and/or release is also mentioned in the definition of EDCs as a mechanism of action. Catecholamine hormones (e.g. norepinephrine) are stored in granular vesicles of chromomaffin cells within the adrenal medulla and within presynaptic terminals in the central nervous system. Therefore, they can be released quickly on demand. In contrast, steroid hormones are not stored intracellularly within secretory granules, but are readily synthesized after gonadotropin stimulation of the gonads.

The formamidine pesticides chlordimeform and amitraz have been reported to block norepinephrine binding to the alpha 2-andrenoreceptors [37]. Norepinephrine is critical for the preovulatory increase in the pulsatile release of GnRH and the subsequent ovulatory surge of LH [38]. Thiram suppresses the proestrus surge of LH and delays ovulation in the female rat [39]. Disruption in the timing of the LH surge could alter the viability and the quality of the oocyte [40] and a potential conceptus by pre-ovulatory over-ripeness ovopathy (PrOO) [41]. Inhibition of progesterone secretion and poor conception occurred after malathion exposure at the onset of estrus in cattle [42].

#### 3. Interference with hormone transport and clearance

For the most part, steroid hormones in the bloodstream do not float around freely, but are bound to carrier proteins, such as SHBG and albumin. Because only free hormones can be biologically active, increases or decreases in the concentration of SHBG will have a major impact on the available and active steroid hormone concentrations in blood. Estrogens are known to increase the synthesis of SHBG in the liver and thus increase the SHBG concentration in plasma, whereas androgens decrease these concentrations [25,43]. Substances that mimic these natural hormones may cause similar changes, but no specific papers dealing with effects of pesticides on SHBG levels have been found.

In contrast, reports are known about the influence of pesticides on clearance of steroid hormones, mostly occurring in the liver. The clearance rate is different for each hormone and is influenced by compounds that alter liver enzyme activity involved in hormone clearance. Many pesticides induce the liver enzymes monooxygenase and UDP-glucuronosyltransferase [44], resulting in increased clearance of the pesticide itself for detoxification purposes, but also of testosterone [45,46]. For instance, DDT analogs are potent inducers of hepatic microsomal monooxygenase activity *in vivo *[47], which degrades endogenous androgens, resulting in suppressed androgen receptor mediated activity. These effects have also been suggested for endosulfan and mirex [48]. Similarly, treatment with lindane has been reported to increase the clearance of estrogens [49].

#### 4. Interference with hormone receptor recognition and binding

**Figure 2:**
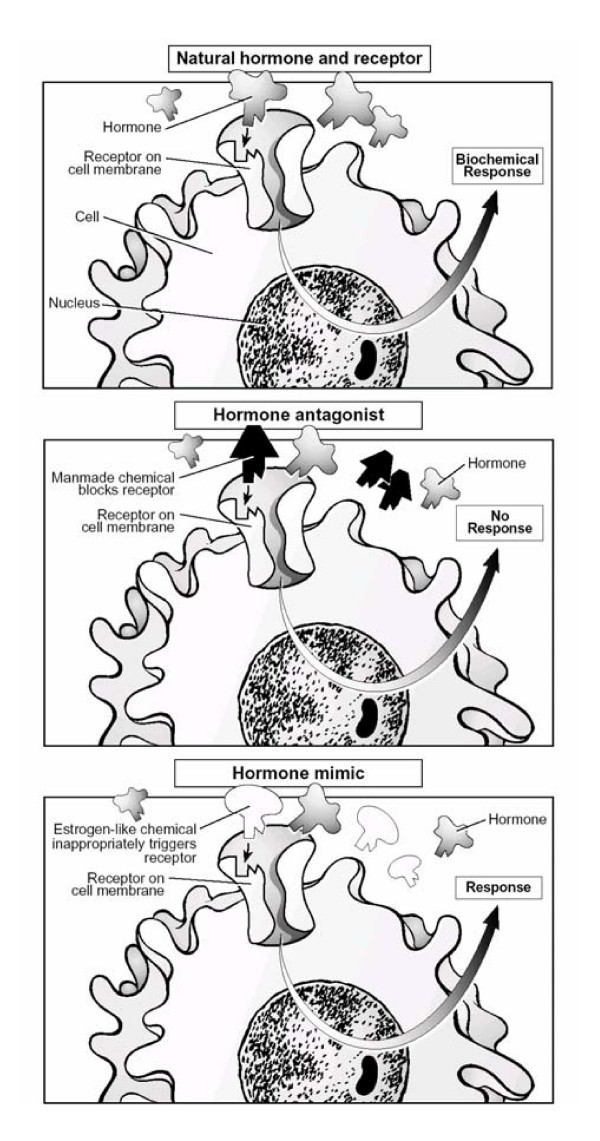
The natural hormone-receptor interaction and the mechanisms of action of hormone antagonists and hormone agonist. (From T. Schettler, Generations at Risk: How environmental Toxicants May Affect Reproductive Health in California, 1995 [23]).

This mechanism of endocrine disruption is much discussed in the literature. Hormones travel from their point of release in the bloodstream to particular tissues where they convey their messages. For the message to be interpreted, hormones bind to receptors. Hormone and receptor have a precise fit, so that only a specific type of hormone can bind to a specific receptor (see Figure 2) [23]. A number of environmental agents may alter this process by mimicking the natural hormone (agonists) or by inhibiting receptor binding (antagonists). The latter mechanism is based on complete or partial blocking of the specific receptor. Regarding the estrogen receptor, this mechanism only applies when the endocrine disruptor concentration is high, because the affinity of endocrine disruptors for the estrogen receptor is usually many times lower than that of 17-beta-estradiol. Three different mechanisms are elaborated below: 1. binding and activating the estrogen receptor; 2. binding without activating the estrogen receptor; and 3. binding other receptors.

#### 4.1 Binding and activating the estrogen receptor

When an endocrine disruptor or one of its metabolites bind and activate the estrogen receptor, the endocrine disruptor will imitate the hormone 17β-estradiol. The substance thus acts as an agonist and is called estrogenic. Possible effects may be decreased production of GnRH by the hypothalamus (negative feedback system) and of LH and FSH by the pituitary gland. As a result, the levels of LH and FSH will drop and finally lead to a lack of estradiol. Under normal circumstances, the hypothalamus will then be triggered to produce more GnRH, but this will be prevented by the endocrine disruptor. As a result, the hormonal cycle may be disrupted.

Several pesticides or their metabolites have been reported to possess estrogenicity *in vivo*, e.g. methoxychlor [50-53], kepone [54], and DDT [55-57] and/or *in vitro*, e.g. o,p'-DDT and p,p'-DDT [57], endosulfan [31,58,59], toxaphene [58-60], dieldrin [31,59-61], fenarimol [31,62], triadimefon [62], triadimenol [62], aldrin [55], endrin [55], methiocarb [31], pentachrophenol [63], nonylphenol [63], alachlor [64], fenvalerate [65], chlordecone [66], and sumithrin [65,66]. Some pesticides are weakly estrogenic, but may act additively in combination of two or more pesticides. When mixed together they may induce estrogenic responses at concentrations lower than those required when each compound is administered alone [60].

#### 4.2 Binding without activating the estrogen receptor

If an endocrine disruptor or its metabolites bind, but do not activate the estrogen receptor, the substance acts as an antagonist and inactivates the estrogen receptor, preventing estradiol to bind. As a result, the hypothalamus stimulates production of GnRH and the pituitary gland will produce more LH and FSH. The concentration of estradiol will also increase, but because the feedback mechanism is disrupted, GnRH will not decrease. Eventually, the estrogen receptors may become less sensitive through prolonged exposure to high concentrations of estrogen and/or endocrine disruptors.

The results of a study by Cooper *et al. *indicate that lindane may effectively block the response of estrogen-dependent tissues and that this apparent anti-estrogenic effect is responsible for the disturbances observed in the neuroendocrine control of ovarian function in rats [67]. Other studies also suggest that lindane is anti-estrogenic and is able to disrupt the estrus cycle [68,69]. Atrazine, simazine, and diaminochlorotriazine expressed anti-estrogenic activity in uteri of female rats without expressing intrinsic estrogenic activity, but the precise mechanism is not known [70,71].

#### 4.3 Binding other receptors

The fungicide vinclozolin and two of its metabolites bind the androgen receptor and act as androgen receptor antagonists *in vitro *and *in vivo *[72-75]. Procymidone and DDT also express anti-androgenic activity [74,76]. A paper by Kelce *et al. *presents consistent evidence that DDT and DDE compete with androgens for their receptors [66]. In addition, several studies suggest that the pesticides methoxychlor [74], linuron [74,77], fenitrothion, and biphenol act as androgen antagonists *in vitro *and/or *in vivo *[74,78-80]. The presence of a potent androgen antagonist in a sufficient internal dose may create an overall estrogenic effect. The pyrethroid insecticides fenvalerate and d-trans allethrin seem to antagonize the action of progesterone [65].

Some endocrine disruptors act through one of the above mechanisms, while others may cause their effects in several different ways. For instance, DDT was found to be estrogenic, but can also bind to the androgen receptor [63]. The pesticides dieldrin, endosulfan, methiocarb, and fenarimol are known as both estrogen agonists and androgen antagonist [31]. Prochloraz acts as estrogen and androgen antagonist [31]. Endosulfan and alachlor have been found to bind to the estrogen receptor as well as the progesterone receptor [81]. Kelce *et al. *showed that chlordecone binds rather efficiently to estrogen receptors while it may also bind to androgen receptors at higher concentrations [66]. Although the potencies of these pesticides to act as hormone agonist or antagonists are low compared to the natural ligands, the integrated response in the organism might be amplified by the ability of pesticides to act via several mechanisms and by frequent simultaneous exposure to different pesticides [31].

#### 5. Interference with hormone postreceptor activation

If an agonist binds to its receptor, a cascade of events is initiated for the appropriate cellular response necessary for signal transduction across the membrane or, in case of nuclear receptors, the initiation of or alteration in DNA-transcription and protein synthesis [25]. Lindane has been demonstrated to decrease phosphatidylinositol turnover in the membrane and to reduce protein kinase-C activation [82]. Steroid hormone receptor activation may also be modified by indirect mechanisms such as a downregulation, which is seen after TCDD exposure [83,84].

#### 6. Interference with the thyroid function

Pesticides like chlorophenols, chlorophenoxy acids, organochlorines, and quinones have been shown to alter thyroid gland function and to reduce circulating thyroid hormone levels [85,86]. Reduction in thyroid hormone levels can compromise the catalytic activity of hepatic cytochrome P450 monooxygenases, resulting in an altered hepatic androgen metabolism [87].

#### 7. Interference with the central nervous system

The central nervous system (CNS) is very important in the integration of hormonal and behavioral activity. Disturbances in these finely tuned mechanisms can severely impair normal adaptive behavior and reproduction. Since many pesticides are known to be neurotoxic, it is conceivable that these chemicals can disrupt the coordinating activity of the CNS by disrupting brain cell functions [25]. Also, pesticides can alter the hypothalamic and pituitary function and thus secretion of GnRH, LH, and FSH in a more direct manner by modifying the feedback of endogenous hormones. For example, it has been demonstrated that low-dose exposure to o,p-DDT and methoxychlor can result in diminished hypothalamic and pituitary function in rodents [88,89]. Finally, it is postulated that any environmental compound mimicking or antagonizing steroid hormone action could presumably alter the glycosylation of LH and FSH, thereby reducing their biological activity [90].

### Potential effects of hormone disruption on the female reproductive system

The function of the female reproductive system depends upon hormone concentrations and their balance. Endocrine disruption may result in disturbances in the reproductive system, such as modulation of hormone concentrations, ovarian cycle irregularities, and impaired fertility [91], which may be due to any of the mechanisms mentioned above. In many studies addressing these disturbances, however, the mechanisms are not specified. These studies, describing the effects of endocrine disrupting pesticides on the female reproductive system in more general terms, are summarized below (see also Table 1). As the majority of these studies are experimental animal studies, one should keep in mind that the estrus cycle in animals only partly corresponds with the ovarian cycle in humans, but that the phases (proestrus, estrus, metestrus, and diestrus) are different. Estrus is the period of greatest female sexual responsiveness usually coinciding with ovulation. Diestrus is the luteal phase of the estrus cycle when the female is not receptive to the male and the progesterone levels are high.

#### Modulation of hormone concentrations

Hormonal balance, i.e. a proper level of sexual hormones, is important to preserve female reproduction and maintain fertility. This balance can be disturbed by changing levels of estrogen or progesterone. Estrogen levels may be decreased by several pesticides. Treatment of rats with the insecticide heptachlor suppressed estradiol concentrations in blood and reduced the production of estradiol by ovarian cells of treated rats [92,93]. Lindane, atrazine, and simazine also cause a decrease in circulating estradiol levels in rats [94,95]. In monkeys, ovulatory levels of estradiol were reduced after high doses of hexachlorobenzene [96], which also induced anovulatory cycles and suppression of circulating levels of estradiol [97], and a dose dependent suppression of serum progesterone concentrations during the luteal phase [98]. Progesterone levels may be decreased by exposure to methoxychlor as well, especially during the estrus phase of the estrus cycle in rats [99,100]. During early pregnancy, progesterone concentrations decreased after treatment with DDT in rabbits [101].

#### Ovarian cycle irregularities

The female ovarian cycle is the result of a balanced cooperation between several organs and is determined by a complex interaction of hormones. Ovarian cycle irregularities include disturbances in the ovarian cycle (e.g. longer cycle, persistent estrus) and ovulation problems (deferred ovulation or anovulation).

##### Disturbances in the ovarian cycle

Organochlorine compounds are known to interrupt the estrus cycle in rats [102-104]. The number of estrus cycles and the duration of each phase of the estrus cycle were significantly affected after treatment of rats with methyl parathion [105,106]. The pesticides dimethoate, malathion, and sumithion gave similar results[107,108] Atrazine, an antagonist of the estradiol receptor, can alter the estrus cyclicity in rats and caused lengthening of the estrus cycle and an increase in the number of days in estrus [109-112]. Carbofuran effected the estrus cycle by showing a decrease in the number of estrus cycles and the duration of each phase [113], which may be due to a direct effect on the ovary or on the hypothalamus-pituitary-ovarian axis causing hormonal imbalance. The pesticide heptachlor may cause disrupted and prolonged estrus cycles [114]. Treatment with DDT and chlordecone resulted in persistent estrus in rats. Lindane induced marked disturbances in the estrus cycle, prolonging the proestrus phase considerably and thereby delaying ovulation [68,69,115]. The pesticides hexachlorobenzene, mancozeb, and 3,3',4,4'-tetrachloroazoxybenzene may also cause cycle irregularities, such as a decrease in the number of estrus cycles and an increase in the duration of diestrus [96,98,116,117].

##### Ovulation problems

Endocrine disruptors with estrogenic properties may be able to block ovulation similar to contraceptive pills. The midcycle surge of LH from the pituitary gland provides the physiological trigger for the process of ovulation in the mammalian female. Any agent that compromises the LH surge could function as a reproductive toxicant [118]. Atrazine, for instance, can cause anovulation due to suppression of LH secretion [119]. The proestrus LH surge in rats was suppressed after treatment with chlordecone [103], whereas Muller *et al. *found that hexachlorobenzene can block ovulation in rhesus monkeys [97]. In this study, low estrogen levels were found during anovulatory cycles. The pesticides thiram and sodium N-methyldithiocarbamate may also inhibit ovulation in rats [33,120].

##### Impaired fertility

Human fertility is a delicate process that can be influenced by many factors, such as hormonal imbalance caused by pesticides [121]. However, in most studies it is not clear whether impaired fertility is due to hormonal imbalance or to other toxic effects. Fenarimol was found to cause a dose-related decrease in fertility in rats [27]. Baliger *et al. *found a decrease in the number of healthy follicles and an increase in the number of atretic follicles in mancozeb treated rats [116]. Also, treatment with methoxychlor and chlordecone caused an increase in the number of atretic follicles. This indicates a potential reduction in fertility [102,104]. A decrease in the number of healthy follicles was also seen after methyl parathion treatment [106]. Exposure to mancozeb and methyl parathion may lead to a decrease in uterus weight as well, which may affect implantation [106,122]. Inhibition of implantation can be caused by mancozeb [122], methoxychlor [100], heptachlor [93], and chlordimeform [123]. Methoxychlor accelerates embryo transport rate in rats and induces preimplantation embryonic loss, perhaps due to this acceleration [124]. The insecticide lindane modifies sperm responsiveness to progesterone *in vitro*, a physiological effect of the acrosome reaction [125,126], which could be a cause of infertility in women exposed to lindane. Female alligators from Lake Apopka polluted with dicofol and DDT exhibited abnormal ovarian morphology with large numbers of polyovular follicles and polynuclear oocytes [127]. Also, their estradiol levels were almost twice as high as in female alligators from a control lake. The investigators suggested that the gonads of juveniles from Lake Apopka were permanently modified in ovo, so that normal steroidogenesis is not possible and normal sexual maturation is unlikely.

### Real-life risks of pesticide exposure on the female reproductive function?

The studies described in the previous paragraphs are mostly studies involving laboratory animals (*in vivo*) or cell cultures (*in vitro*). Animal and *in vitro *studies are widely used and are often the first indicators of potential reproductive or developmental effects. However, the health risks for human populations may be considerably different, because of differences in exposure levels [128], reproductive issues, metabolism, size, and lifespan, which make it difficult to extrapolate from effects found in animals to effects that might be expected in women [23]. Recognising that a pesticide has the potential to cause harm reveals only a hazard. The risk of this pesticide actually inducing a biological effect depends on its properties, but the effect will only occur when exposure reaches a particular level [128]. Endocrine disruptors that accumulate in the body may eventually reach higher threshold levels necessary for exertion of their biological effects. Throughout the intricate processes of the menstrual cycle, ovum production, fertilization, implantation, and growth and developmental of the fetus, specific and often short time intervals exist in which these processes may be particularly susceptible to low-dose exposures of endocrine disruptors [23].

Another difficulty in human studies is that people can be exposed to endocrine disruptors in various ways, such as through iatrogenic exposure, endogenous estrogens, natural substances with estrogenic or androgenic activity (bioflavonoiden), and environmental endocrine disruptors like pesticides. In addition, it is quite feasible that interactions between endocrine disruptors play a role when there is combined exposure [129]. Therefore, the results of epidemiologic studies seldom pertain to specific pesticides and firm conclusions about causality of effects of endocrine disrupters on the female reproductive system are lacking. Still, we will give a quick overview of the epidemiological studies which found associations between pesticide exposure and reproductive effects that may be due to disruption of the female hormonal function (see Figure 1).

#### Menstrual cycle disturbances

Two studies examined the effects of pesticide exposure on the menstrual cycle. Both found associations between serum levels of DDT or a metabolite of DDT and short cycles [130] and undefined 'menstrual disturbances' [131]. A recent study observed that women who currently used pesticides experienced longer menstrual cycles and increased odds of missed periods compared with women who never used pesticides [10]. In addition, women who used probably hormonally active pesticides had a 60–100% increased odds of experiencing long cycles, missed periods, and intermenstrual bleeding compared with women who had never used pesticides.

#### Infertility

In a study in the USA, infertile women were observed to be three times more likely to ever having been exposed to pesticides [14] and nine times more likely to ever having worked in agriculture [13]. Another study found no correlations between infertility and self-reported overall pesticide exposure, working in the agricultural sector, or living on a farm during the two years before the diagnosis of infertility or the last pregnancy [132]. However, an association was present when exposure to herbicides only was considered.

#### Time-to-pregnancy

Three studies examined the effects of pesticide exposure on the time it took couples to become pregnant [time-to-pregnancy (TTP)], which is affected by disturbances in the whole chain from gametogenesis to embryonic survival [133,134]. No consistent pattern of associations was observed by Curtis *et al. *in Canada, but some specific pesticides were tentatively associated with prolonged TTP [135]. Abell *et al. *and Idrove *et al. *found significantly prolonged TTPs related to high levels of pesticide exposure among female worker in flower greenhouses [11,12].

#### Spontaneous abortion/stillbirth

A number of studies reported that among women occupationally exposed to pesticides and/or working in the agricultural sector the risks of spontaneous abortion [136-139] and stillbirth [140-144] seemed to be significantly increased. In addition, two reviews concluded that there are numerous indications that exposure to pesticides may contribute to spontaneous abortion and/or stillbirth [145], but it is unclear whether this should be considered as an endocrine disrupting effect [146].

#### Developmental defects

For birth defects, an overall association with agricultural work was observed in a large cohort study [147]. Studies focusing on specific birth defects found associations between agricultural work and orofacial clefts [138], hypospadias [148], total anomalous venous return [149], spina bifida [148,150], and limb reduction defects [148,151], although the relation with limb reduction defects was contradicted by one other study [152]. In a well-conducted Finnish study of women in agricultural occupations, the investigators found that exposure to pesticides during the first trimester of pregnancy nearly doubled the risk of cleft lips and palates in offspring [153]. A slightly increased risk for central nervous system defects was also observed. Again, a cause-effect relation between these defects and exposure to endocrine disrupting pesticides could not be established.

## Conclusion

In this review, we described the different ways in which pesticides may disrupt the hormonal function of the female reproductive system and in particular the ovarian cycle. Pesticides are not one common substance, but comprise a large number of distinct substances with dissimilar structures and diverse toxicity which may act through different mechanisms. Therefore, it is most likely that not just one but several of the above-mentioned mechanisms are involved in the pathophysiological pathways explaining the role of pesticide exposure in ovarian cycle disturbances ultimately leading to fertility problems and other reproduction toxic effects. A disadvantage of the studies described is that they were mostly laboratory animal and cell culture studies. These often provide the first indications of potential reproductive effects of a chemical, but it is difficult to extrapolate the effects found in laboratory animals to effects that might be expected in women. Therefore, we also reviewed epidemiological studies which lead to the conclusion that exposure to pesticides may be associated with menstrual cycle disturbances, reduced fertility, prolonged time-to-pregnancy, spontaneous abortion, stillbirths, and developmental defects. However, in most of these studies specific information on pesticide exposure and the pathophysiological mechanisms involved was missing. Furthermore, we have to take into account that dose, timing, and duration of exposure are critical to the ability of a pesticide to cause harmful effects. Nevertheless, real-life occupational exposures to pesticides appear to have adverse effects on female reproduction. In future research, information on the ways in which pesticides may disrupt the hormonal function as described in this review, can be used to generate specific hypotheses for studies on the effects of pesticides on the ovarian cycle, both in toxicological and epidemiological settings.

## Abbreviations

CNS central nervous system

DDE dichlorodiphenyldichloroehthylene

DDT dichlorodiphenyltricloroethane

EDC endocrine disrupting chemical

EPA environmental protection agency

FAO food and agriculture organization

FSH follicle stimulating hormone

GnRH gonadotropin releasing hormone

LH luteinizing hormone

PCB polychlorinated biphenyl

SHBG sex-hormone-binding globulin

SMD sodium N-methyldithiocarbamate

PrOO pre-ovulatory over-ripeness ovopathy

TTP time-to-pregnancy

WHO world health organization

## Competing interests

The author(s) declare that they have no competing interests.

## Authors' contributions

RB performed the literature search and wrote the first draft of the manuscript. All authors contributed substantially to the conception of the outline of the review, gave advise during the literature study, and critically revised subsequent versions of the manuscript. All authors read and approved the final manuscript.
